# Disease and Insanity

**Published:** 1899-08-12

**Authors:** 


The Hospital

A Journal of

The Medical Sciences and Hospital Administration.

Vol. XXVI.—No. 672.] Edited by Sir Henry Burdett, K.C.B., and Solomon C. Smith, M.D., M.R.C.P. [August 12, 1899.

### Disease and Insanity.

The last few days have witnessed the publication of the ordinary annual report of the Commissioners in Lunacy, and also of the ordinary quarterly report of the Registrar-General. The two, taken together, do not constitute pleasant reading; for, while the latter tells us of a death-rate less by 1·1 annually per thousand persons living than that of the average of the second quarter for ten years previously, the former tells us of an increase of no less than 3,114 in the insane population of England and Wales, as compared with the year 1898, being the largest which has hitherto been recorded within the same period of time. In other words, the deaths of the second quarter of the year have been fewer by about 8,000 than the customary average; while, taking the same period of ten years for the purposes of comparison, the insane have increased from one person in 337 of the population to one in 302. Contemporaneously with an increase of longevity which points conclusively to an improved condition of national health, we have an increase which can only be described as alarming in the proportion of persons whose mental faculties give way under the stress of the demands of life. The increase is occurring, moreover, during a time of almost unexampled prosperity, and cannot be referred to the effects of privation or distress. Whatever may be its causes, it imperatively demands the attention of physicians, of philanthropists, and of statesmen.

It would, of course, be impossible to define insanity; but there can be no doubt that, in its declared forms, it is a consequence of disease, and we are therefore constrained to admit that there is at least one form of disease which is gaining ground amongst us, and which no modern discoveries have so far rendered more amenable to treatment. People differ in their mental attributes and capacities just as they differ in their strength and co-ordination of their muscles. There cannot, in the nature of things, be any inflexible standard for either. We have to recongnise that the intellect of the philosopher, or the strength of the athlete, or the dexterity of the eminently skilful manipulator, is something primarily dependent upon structure, although probably in most cases, if not in all, enhanced by diligent cultivation. In all these respects we see gifted individuals who surpass the common average of mankind, and in all of them we see individuals the reverse of gifted who fall below that average. There is no line of demarcation; but, practically, there is no difficulty in deciding when weakness or awkwardness of muscle passes into inco-ordination or into paresis. In like manner we can point to insanity, although we cannot define it. We recognise, in a rough and ready sort of way, what degree of deviation from the mental operations of the average man renders the person so deviating unfit to manage his affairs, or to mix on terms of equality with his fellows. When the lunatic dies we find evidences of structural degeneration in his nervous centres, just as we should do in the nervous centres of a paralytic. We are compelled to recognise the course of a morbid process, which we can trace by its effects upon function during life, and by its effects upon structure after death. Beyond these we cannot be said to possess any knowledge with regard to it. Since the time of Conolly, who was once described by Charles Dickens as "the wisest of the *mad* doctors," few of the estimable gentlemen who have been in charge of asylums have displayed any great development of what may be called the clinical instinct; and their contributions to the study of insanity have greatly consisted in the invention of jargon. It is considered the correct thing to describe Christopher Columbus as a "paranoiac," a coinage which conveys no more information than if we called him a "crank," and which is in every way inferior to the simple and stately English of the New Testament, "Paul, thou art *beside thyself*." The invention of jargon is, perhaps, the most barren of human occupations. Several years ago a committee of the first London County Council, after taking much evidence, issued a report in which they strongly recommended the establishment of a "hospital" for the insane, as distinguished from an "asylum"—an institution of moderate size, under the care of visiting physicians, who were to have no responsibilities as to the expenses or household management, and who were to regard the inmates simply as sick persons, who were, if possible, to be cured of diseased conditions prejudicially affecting their aptitude for correct thinking. The proposal was at once too "advanced" and not sufficiently "progressive" to find favour with the Council, who have since fancied they were taking a step in the indicated direction by the appointment of a "pathologist"—a learned and skilful gentleman, whose function it will be, after the steed is stolen, to call attention to any matters which the thieves may have left behind them in the stable. The pathologist is admirable; but what is really wanted is the trained and skilful clinical observer, whose mind is not distracted from his duty as a physician by the burden of responsibilities wholly foreign to his calling.

